# Head ultrasound, CT or MRI? The choice of neuroimaging in the assessment of infants with congenital cytomegalovirus infection

**DOI:** 10.1186/s12887-019-1562-z

**Published:** 2019-06-05

**Authors:** Mina Smiljkovic, Christian Renaud, Bruce Tapiero, Valérie Lamarre, Fatima Kakkar

**Affiliations:** 1 Department of Pediatrics, Division of Infectious Diseases, The Hospital for Sick Children, University of Toronto, Toronto, ON Canada; 2 Department of Microbiology and Infectious Diseases, CHU Sainte-Justine, Université de Montréal, Montréal, QC Canada; 3 Department of Pediatrics, Division of Infectious Diseases, CHU Sainte-Justine, Université de Montréal, 3175 Côte Sainte-Catherine, Montreal, QC Canada

**Keywords:** Cytomegalovirus, Infection, Congenital, Pediatrics, Imaging

## Abstract

**Background:**

Despite growing interest in universal screening for congenital CMV infection (cCMV), and data to support treatment for cases with central nervous system (CNS) involvement, there is limited regarding the optimal imaging modalities to identify CNS involvement. The objective of this study was to assess the concordance between head ultrasound (US) and magnetic resonance imaging (MRI) or computed tomography (CT), in identifying neurological abnormalities in infants with cCMV infection, and to determine whether the addition of advanced neuroimaging after US had an impact on clinical management.

**Methods:**

Retrospective review of infants with cCMV infection, referred to the *Centre d’Infectiologie Mère-Enfant (CIME)* at Sainte-Justine Hospital Center in Montreal, between 2008 and 2016. Only patients who underwent head US followed by and brain MRI or CT scan were included in this analysis.

**Results:**

Of 46 cases of cCMV identified during the study period, 34 (74%) had a head US followed by MRI (*n* = 28, 61%), or CT scan (*n* = 6, 13%). In the majority of cases (*n* = 24, 71%), both images were concordant (11 both reported abnormal, 13 both reported normal). In 5 cases, US was reported normal and subsequent imaging (MRI = 4, CT = 1); reported abnormal. In all 5 cases patients were clinically symptomatic and met treatment criteria even in the absence of neuroimaging findings. In 5 cases, US was reported abnormal with a subsequent normal MRI (4) or CT (1); in 2 of these cases, patients were clinically symptomatic and met treatment criteria regardless of neuroimaging findings. However, in 3 cases, the patients were clinically asymptomatic, and in 2 of these cases, treated based only on the abnormal US findings.

**Conclusions:**

In this study, we found that that sequential US and MRI were concordant in the majority (71%) of cases in detecting abnormalities potentially associated with cCMV infection. While the addition of MRI to baseline head ultrasound did not influence the decision to treat in clinically symptomatic infants, the addition of MRI to infants with abnormal HUS imaging who are clinically asymptomatic could help refine treatment decisions in these cases.

## Background

Congenital CMV (cCMV) infection is the most common cause of intrauterine viral infection [1]. Affecting approximately 1 in 150 live births, it represents a major cause of sensorineural hearing loss (SNHL) and neurodevelopmental delay among children worldwide [2]. Despite growing interest in universal screening for congenital CMV infection (cCMV), a paucity of data exists regarding the optimal imaging modalities to identify central nervous system (CNS) involvement. While studies have examined the value of prenatal neuroimaging for infants with cCMV [3, 4], a standard of care for postnatal imaging of the CNS of children evaluated for cCMV has not been established. A recent expert consensus recommended antiviral treatment for infants with evidence of CNS disease, but did not provide guidance regarding preferred modalities to identify CNS abnormalities [1]. Therefore, current practices vary according to center-based protocols or physician expertise [5], and may include head ultrasound (US), brain magnetic resonance imaging (MRI), computed tomography (CT), or combinations thereof.

With a growing interest in cCMV screening programs, a better understanding of the role of different neuroimaging modalities is necessary, given that abnormal CNS findings represent a criteria for treatment. While CT is most sensitive at detecting calcifications, the associated radiation exposure has led many to favor US as the primary neuroimaging modality, supported by recent improvements in ultrasound technology and increased ability to detect calcifications [6]. However, MRI may be better at detecting white matter changes, gyration abnormalities and cerebellar hypoplasia [7]. At present, there is no clear preferred imaging modality, and local accessibility, cost-effectiveness and radiation exposure are factors taken under consideration by individual physicians. In response, the primary objective of this study was to assess the concordance between US and MRI in identifying neurological abnormalities in infants with cCMV infection, and to determine whether the addition of advanced neuroimaging after US had an impact on clinical management.

## Methods

### Study design

We performed a retrospective study of infants with cCMV infection who were referred to the *Centre d’Infectiologie Mère-Enfant (CIME)* at the Centre Hospitalier Universitaire Sainte-Justine (CHUSJ), a tertiary pediatric care center in Montreal, from January 2008 to December 2016. Case detection was performed via the microbiology laboratory clinical database and defined based on positive CMV testing within the first 21 days of life, with the following; culture or shell vial assay on urine or saliva, and qPCR in blood, urine, saliva or cerebrospinal fluid (CSF). All positive saliva tests were confirmed with urine or blood assays. The study was approved by the CHUSJ Research Ethics Board (Study number: 20171282).

Statistical analysis was performed using STATA statistical software v.14.1 (Stata Statistical Software*:* Release 15. College Station, TX. Statacorp LP). Patient characteristics were compared using Chi-square tests or Wilcoxon rank-sum where appropriate. Means and standard deviations were used to describe continuous variables and proportions to describe categorical variables.

### Neuroimaging and clinical classification

Neuroimaging was performed routinely at the baseline evaluation (at time of diagnosis, in the first 21 days of life) of all infants with cCMV, and the choice was at the discretion of the treating physician. Only patients who underwent sequential US and MRI or CT were included in this analysis. Ultrasound over the anterior fontanel was performed by a pediatric radiologist. Subsequent imaging (CT or MRI) was at the discretion of the treating physician, or at the recommendation of the radiologist following ultrasound findings. Multi-detector scanners were used for computed tomography (CT) without sedation, using non-ionic iodinated contrast agents. Contrast enhanced MRI was performed with sedation (IV Pentobarbital) for all infants. Standard protocols included sagittal and axial T1 weighted images, axial T2, coronal FLAIR and axial diffusion weighted imaging. Non-ionic Gadolinium based contrast agents were used and 3D T1 weighted sequence was acquired after the injection. The neuroimaging results were categorized as normal or abnormal based on the absence or presence of any findings suggestive of cCMV infection, as reported to the treating physician by pediatric radiologists aware of the diagnosis of cCMV infection. The following findings were considered abnormal: intracerebral calcifications, lentriculostriate vasculopathy, periventricular leucomalacia, ventriculomegaly, cortical or cerebellar malformations, migration defects, cystic abnormalities, and white matter abnormalities.

Routine baseline evaluation for all infants included audiology and ophthalmology assessments, blood work (complete blood count, liver function tests, and bilirubin) and detailed physical examination by a pediatrician. Infants were considered symptomatic if they had one or more of the following: persistent thrombocytopenia (more than one platelet count of < 100,000/uL), petechiae, hepatomegaly, splenomegaly, hepatitis (raised transaminases), conjugated hyperbilirubinemia, systemic signs such as intrauterine growth restriction (IUGR), central nervous system involvement such as microcephaly, radiological abnormalities consistent with CMV central nervous system disease (see above), chorioretinitis, sensineural hearing loss (SNHL), abnormal cerebrospinal (CSF) fluid indices for age, or the detection of CMV DNA in CSF fluid.

## Results

Forty-six cases of cCMV infection were identified during the study period. The majority (*n* = 27, 58.7%) were diagnosed at birth because of generalized symptoms consistent with cCMV infection, or failed newborn hearing screen (*n* = 5, 10.1%). The remaining were tested as part of targeted screening of newborns of HIV -infected mothers (*n* = 2, 4.4%), or following suspected maternal seroconversion during pregnancy (*n* = 12, 21.7%).

Of the 46 patients, 34 (74%) underwent sequential baseline imaging, with US followed by MRI (*n* = 28, 61%) or CT (*n* = 6, 13%). Only 2 patients had a CT as a primary study, followed by MRI. The remaining 10 had a single imaging modality (US, CT or MRI). Among cases with sequential imaging, US was performed at a median of 7 days (range 0–61 days) and MRI at a median of 42 days of age (range 2–156 days). CT was performed at a median of 6 days (range 8–21 days). Table 1 describes patient characteristics according to the initial choice of neuroimaging (US vs. advanced modality first). Overall, 39 (85%) had an US first, or as their only imaging modality, while 6 (15%) had either CT or MRI performed first, or as their only neuroimaging. Those who had MRI or CT as their initial choice of imaging were more likely to have SNHL at time of diagnosis (85.7% vs. 33%, *p* < 0.01) and laboratory abnormalities (57.1% vs. 46%), though not statistically significant (*p* = 0.59).

**Table 1:** Patient characteristics according to initial choice of neuroimaging

	Overall	Head US first (or sole imaging modality) (*n* = 39)	CT or MRI first (or sole imaging modality (*n* = 7)	p
Birthweight (g) (mean + SD)	2707 ± 741	2738 ± 791	2540 ± 405	0.54
Gestational age (weeks) (mean, SD)	38.0 ± 2.79	38.0 ± 0.98	38.2 ± 3.02	0.85
Presence of clinically apparent symptoms^a^	24 (54%)	20 (57.1%)	4 (51.2%)	0.78
Microcephaly	5 (11%)	4 (12%)	1 (14%)	0.89
Laboratory abnormalities^b^	22 (48%)	18 (46.1%)	4 (57.1%)	0.59
Sensineural hearing loss^**c**^	19 (41.3%)	13 (33%)	6 (85.7%)	< 0.01
CMV tested on clinical suspicion vs. screening program	28 (61%_)	24 (57.1%)	4 (61.5%)	0.83

^a^ Clinically apparent symptoms: Microcephaly, IUGR, hepatomegaly, splenomegaly, petechiea or purpura, jaundice

^b^ Laboratory abnormalities: Any of persistent thrombocytopenia, hepatitis, hyperbilirubinemia

^**c**^ Defined as a unilateral or bilateral hearing threshold of > 40 dB for at least 2 of the frequencies tested, using a combined protocol of automated distorsion product otoacoustic emissions (DPOAE-A) and automated auditory brainstem response (A-ABR), followed by brainstem auditory evoked potentials by three weeks of age

The most common abnormal neuroimaging findings on US were cystic abnormalities in 31% of cases, followed by intracranial calcifications in 13%, ventriculomegaly and lenticulostriate vasculopathy, each seen in 11% of cases. The most common findings on MRI were white matter abnormalities seen in 16%, followed by cystic abnormalities in 13%, followed by intracranial calcifications in 4% and periventricular leukomalacia in 2% of cases. Most common abnormal findings on CT scan were intracranial calcifications (33%), white matter abnormalities (33%), followed by periventricular leukomalacia and ventriculomegaly in 16% of cases.

Among cases with sequential baseline imaging, the majority (*n* = 24, 71%) were concordant (11 both reported abnormal, 13 both reported normal). In 5 cases, US was reported normal and subsequent MRI (4) or CT (1) abnormal. In all 5 cases, patients were clinically symptomatic and met treatment criteria even in the absence of neuroimaging findings (See Table 2, clinical presentation and neuroimaging results for cases 1–5). In 5 other cases, US was reported abnormal with a subsequent normal MRI (4) or CT (1). In 2 of these cases, patients were clinically symptomatic, with hearing loss (case 6), and hearing loss with systemic symptoms (case 7), and would have been treated per local treatment criteria regardless of neuroimaging findings. However, in 3 other cases (cases 8,9,10), patients were clinically asymptomatic other than the neuroimaging abnormalities. In two cases (8 and 9) cases, patients were categorized as symptomatic based on the abnormal US findings and treated; treatment, once started, was not discontinued after MRI results. Case 10 was an HIV- exposed infant, and due to the need for antiretroviral therapy for HIV prophylaxis and the high risk of toxicity from potential concurrent valganciclovir therapy, a urgent CT scan was performed. This patient was not treated on the basis of the normal CT results.

**Table 2:** Cases with discordant sequential imaging findings

Case	1	2	3	4	5	6	7	8	9	10
HUS	Normal	Normal	Normal	Normal	Grade I intraventricular hemorrhage	Nonspecific hyperechogenicity along the ventriculostriate vessels,with two small periventricular cystic formations	Increase in size of the germinal matrix, with small cystic formations, and lateral ventricle ectasia	Multiple cysts in the germinal region and hyperechogenicity of the thalamostriate vessels	Discrete ventricular asymmetry with a prominence of the right lateral ventricle and small sub ependymal cysts	Cystic abnormalities
MRI or CT	MRI: Nonspecific isolated signal anomaly in the left striatum	MRI: Mild subcortical white matter anomaly in the left parietal lobe	MRI: Anterior and inferior temporal cystic formations	CT: Lentriculostriate vasculopathy	MRI: Abnormal signal in the temporal horns and peritrigonal white matter	MRI:Normal	MRI: Normall	MRI: Normal	MRI: Normal	CT: Normal
Other Symptoms^a^	2,5, 7	1,6	1,2,3,4,5	1,2,3, 4,5,6,8,9	2,3,6	3	1,2, 3,4	None	None	None

^a^Symptoms: Intrauterine growth retardation^1^, persistent thrombocypotenia^2^, sensineural hearing loss^3^, hepatitis^4^, hyperbilirubinemia^5,^, prematurity^6^, systemic symptoms (poor feeding, lethargy, poor weight gain) ^7^, petechiae or purpura^8^, hepatomegaly or splenomegaly^9^

The association between baseline clinical findings and any neuroimaging abnormality is described in the Table 3. For those with discordant results on sequential imaging, CT or MRI results were considered for classification as normal or abnormal. Overall, 61% of all patients had abnormal neuroimaging. Those with laboratory abnormalities were more likely to have abnormal neuroimaging findings (66.7% vs 27.8%, *p* = 0.03) as were those with baseline SNHL (53.5 vs 27.8%), though not statistically significant (p-0.09).

**Table 3:** Clinical findings and neuroimaging abnormalities (HUS, CT, or MRI)

	Normal (*n* = 18)	Abnormal (*n* = 28)	p
Sensineural hearing loss^a^	5 (27.8%)	15 (53.3%)	0.09
Chorioretinitis	0	1 (6.7%)	NA
Microcephaly	2 (13.3%)	3 (12%)	0.90
Clinically apparent symptoms^b^	7 (38.9%)	19 (60.7%)	0.14
Laboratory abnormalities^c^	5 (27.8%)	17 (66.7%)	0.03
Prematurity (< 37 weeks gestational age)	16 (88%)	21 (77.7%)	0.43
Baseline viral load (qPCR) (copies/ml)	69,104	98,040	0.17

^a^Defined as a unilateral or bilateral hearing threshold of > 40 dB for at least 2 of the frequencies tested, using a combined protocol of automated distorsion product otoacoustic emissions (DPOAE-A) and automated auditory brainstem response (A-ABR), followed by brainstem auditory evoked potentials by three weeks of age

^b^Clinically apparent symptoms: Microcephaly, IUGR, hepatomegaly, splenomegaly, petechiea or purpura, jaundice

^c^Laboratory abnormalities: Any of persistent thrombocytopenia, hepatitis, hyperbilirubinemia

## Discussion

In this study, we found that that sequential US and MRI or CT were concordant in the majority (71%) of cases in detecting abnormalities potentially associated with cCMV infection. Among discordant cases, these results had an impact on clinical management, but only among patients who had no other symptoms that met treatment criteria. Specifically, we found that normal US during the postnatal assessment was followed by abnormal MRI or CT in 15% (5/28) of cases who underwent sequential imaging, however these children were all clinically symptomatic and these findings did not influence the decision to treat, given that our threshold for treatment was low. However, this may not be the case across all centers, where strict application of consensus criteria [1, 8] without performing an MRI may miss children who would benefit from treatment. For example in case 2, treatment was initiated due to isolated IUGR, which could be a manifestation of systemic cCMV infection [9], however, not universally recognized as a criteria for treatment. In this case, adherence to consensus criteria and relying on HUS alone would have missed neurological disease in this child, resulting in a missed opportunity for treatment. These results suggest that in infants suspected neurological involvement or neurological abnormalities by exam, MRI should be performed primarily.

Among discordant cases of abnormal US followed by normal MRI or CT (5/34), in 2 of these cases (8 and 9), the decision to treat was based solely on US findings of cystic formations and lenstriculostriate vasculopathy in the absence of other clinical findings. Treatment, once initiated, was not stopped after MRI results given delays in obtaining MRI, resulting in unnecessary treatment for otherwise asymptomatic newborns in 6% of all cases overall (2/34). Case 6 was asymptomatic with isolated SNHL. While some centers, including our own, recommended treatment for these patients, if applying the consensus criteria (which does not currently recommend treatment for asymptomatic patients with isolated SNHL), this patient would have also been unnecessarily treated if relying solely on HUS findings. In short, in otherwise asymptomatic infants or those with isolated SNHL with mild abnormalities on HUS, early advanced neuroimaging within the first month of life is essential to determining the need for treatment.

Although neuroimaging clearly plays an important role in the assessment of affected children, interpretation can be challenging given the wide spectrum of brain abnormalities which are often nonspecific and may be difficult to measure [10]. Ventriculomegaly, diagnosed on US in 2 patients (cases 8 and 9) whose MRIs were subsequently reported as normal, can be difficult to diagnose, with measurements of ventricular diameter that can differ significantly between sonographers, and disagreement with MRI in up to 11% of cases [11]. Moreover, in our study, cystic abnormalities were the most frequent finding, seen in 37% of patients overall, in contrast to cerebral calcifications which are more commonly reported in the literature, occurring in 34–91% of cases [6, 8] and ventriculomegaly, seen up to 45% of cases [3, 10]. Intracranial cysts, though potentially a stigma of cCMV infection, are also reported in the general population, with a prevalence ranging from 0.5–5.2% [12, 13]. Therefore, it is not clear if these findings on HUS represent true sequelae of cCMV infection, or a normal variant. In all 5 discordant cases of abnormal US followed by normal MRI or CT in our study, cystic abnormalities were present on US but in association to other findings (Table 2). Further study is necessary to determine the significance of these mild abnormalities on HUS.

To our knowledge, this is the first series to examine the impact of sequential postnatal US and MRI or CT on the clinical management of children cCMV infection, following published recommendations for the antiviral therapy of infants with CNS disease. Nonetheless, this study has limitations, including the retrospective design and small number of cases. Moreover, the majority (61%) of our patients were diagnosed because of symptomatic disease, rather than through a screening program, therefore the sample may not represent the spectrum of disease within the general population. Our results may only reflect findings of more severe cases, where physicians choose to perform advanced neuroimaging following US. Indeed in this study, physicians were more likely to order CT or MRI first in the presence of SNHL at baseline, suggesting a bias towards advanced neuroimaging in more severe cases.

Finally, the applicability of our findings with respect to clinical management may not be generalizable, as different thresholds exist among centers for the level of symptoms at which cCMV infection should be treated, even with the current consensus guidelines. The treatment of isolated IUGR, or isolated SNHL, is not recommended [1, 8], however many centers in Canada, including own, treat such cases [14]. In our center, of the 4 discordant cases of normal US followed by abnormal MRI, the cases (1–4) met the threshold for treatment. However, this may not be the case elsewhere where treatment is not recommended for isolated IUGR or asymptomatic cCMV infection with isolated SNHL.

## Conclusion

The results of this study suggest that while there may be a discordance in findings from both neuroimaging modalities, the addition of MRI to US in the early assessment of symptomatic children with cCMV infection did not yield additional information to influence the decision to treat, given that our threshold for initiating treatment was low. However, in mildly symptomatic cases where treatment is not indicated according to recent consensus guidelines, or in otherwise asymptomatic children with an abnormal US suggesting CNS disease, the addition of MRI in the neonatal period may help refine treatment decisions.

## Data Availability

The datasets used and/or analysed during the current study are available from the corresponding author on reasonable request.

## Abbreviations

cCMV: Congenital CMV infection
CT: Computer tomography
MRI: Magnetic Resonance Imaging
SNHL: Sensineural hearing loss
US: Head Ultrasound
